# Sintilimab, a PD-1 Inhibitor, Completely Reversed Rarely Refractory Hypofibrinogenemia in a Gastric Cancer Patient: A Case Report and Review of the Literature

**DOI:** 10.3389/fonc.2020.526096

**Published:** 2020-10-19

**Authors:** Shuzhen Ma, Qi Dang, Yali Yang, Yongliang Liu, Yuping Sun, Meili Sun

**Affiliations:** ^1^Department of Oncology, Jinan Central Hospital Affiliated to Shandong University, Jinan, China; ^2^Department of Oncology, Central Hospital Affiliated to Shandong First Medical University, Jinan, China

**Keywords:** sintilimab, immunotherapy, hypofibrinogenemia, gastric cancer, case report

## Abstract

While cancer is often related to hyperfibrinogenemia, it is rarely related to hypofibrinogenemia. Specifically, gastric cancer concomitant with unprovoked hypofibrinogenemia and the corresponding treatment approach have been rarely reported. We presented a case of gastric cancer in a 78-year-old Chinese woman in whom sudden, unprovoked refractory hypofibrinogenemia had been found during the whole brain radiotherapy despite stable clinical condition. Fibrinogen supplementation was not useful for controlling her level of fibrinogen. However, when she received sintilimab, an immunotherapy drug acting as programmed death receptor 1 inhibitor, to treat her gastric cancer, fibrinogen rose to the normal level. We also reviewed the literature to explore the causes of hypofibrinogenemia in tumor patients. This case suggests that we need to pay attention to tumor-related coagulation disorders, and monitoring coagulation indicators is essential. Treating primary disease by immunotherapy drugs may be an important method to improve the level of coagulation factors. This is the first report of sintilimab reversing a rare refractory hypofibrinogenemia in a patient with gastric cancer.

## Background

Gastric cancer is one of the most common gastrointestinal malignancies worldwide and also the second leading cause of cancer-related mortality in China (1). Gastric cancer has always been a major clinical challenge due to poor prognosis and inefficient treatments. Currently, radiotherapy, chemotherapy, surgery, or their combinations are used in cancer therapies, while each of such treatments has both advantages and disadvantages. The emergence of nanomedicine and immune checkpoint inhibitors, such as cytotoxic T-lymphocyte antigen 4 (CTLA-4) and programmed death receptor 1 (PD-1), has revolutionized the treatment of solid tumors (2), which may improve the quality of life and extend the survival rate of patients with gastric cancer.

The universal consensus is that malignancy is one of the highest risk factors for hypercoagulability of blood, driven in part by the release of procoagulant factors and reduced fibrinolytic activity (3). However, a sudden, extreme deficiency of fibrinogen, the clotting factor, and subsequent subcutaneous hemorrhage caused by hypocoagulability occurred in our case. Fibrinogen is a glycoprotein synthesized in hepatocytes, and it functions in the final steps of blood coagulation cascade as a precursor monomer of the fibrin hemostatic plug. Recently, fibrinogen has been shown to participate in tumorigenesis, formation of stroma, angiogenesis, and tumor metastasis (4). Therefore, the relationship between hyperfibrinogenemia and cancer attracts more attention than cancer-related hypofibrinogenemia. The decrease in fibrinogen may occur due to synthesis disorder, increased consumption, or hyperfibrinolysis. Hypofibrinogenemia can lead to spontaneous bleeding. Currently, the main method of fibrinogen supplementation is infusion of fibrinogen concentrate, cryoprecipitate, and fresh frozen plasma (5).

## Case Presentation

**FIGURE 1:**
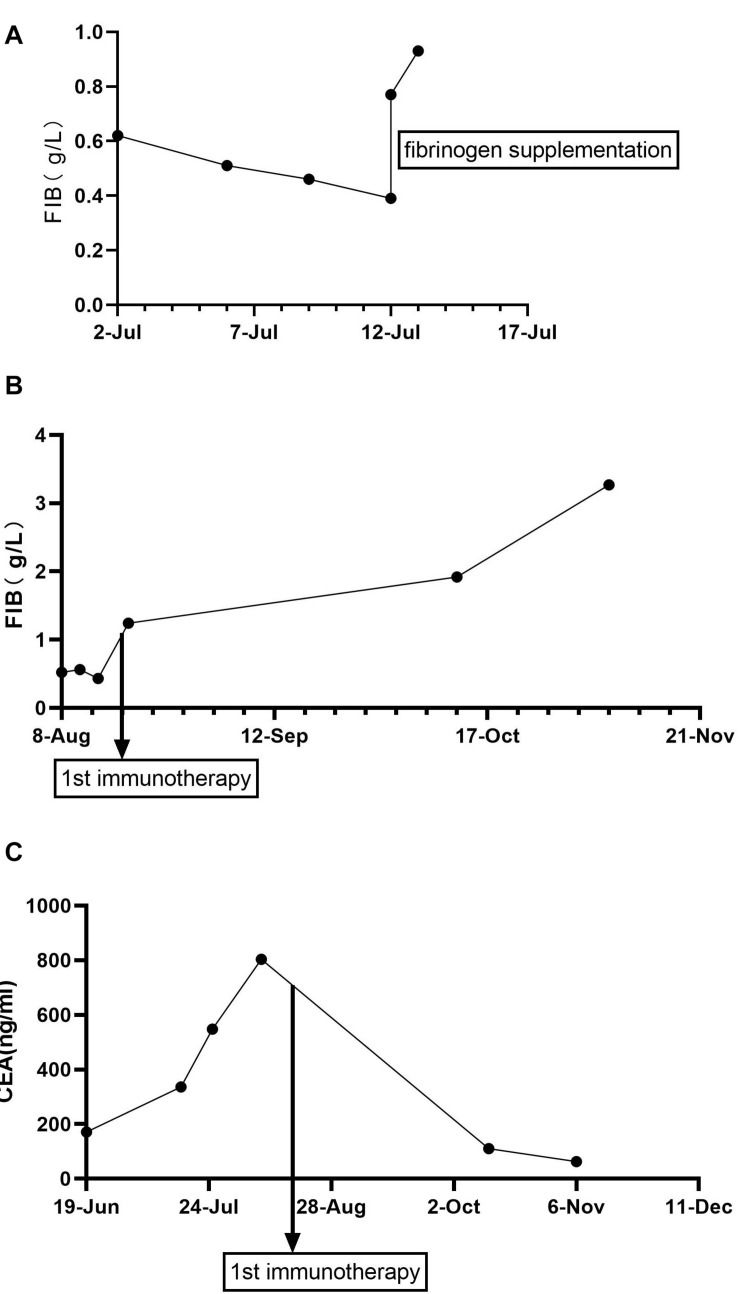
Fibrinogen and CEA levels during treatment (g/L). **(A)** Fibrinogen levels during WBRT. **(B)** Fibrinogen levels during immunotherapy. **(C)** CEA levels (ng/mL) during treatment. Fibrinogen normal value, 2.00–4.00 g/L; the critical value, <1 g/L; CEA normal value, <5 ng/mL. CEA, carcinoembryonic antigen.

Here, we present a case of gastric cancer concomitant with hypofibrinogenemia. A 78-year-old woman visited a hospital in Linyi City in August 2018 and complained of swelling of the right lower limb. On further workup, venous thrombosis was diagnosed and anticoagulant therapy was applied. During hospitalization, screening of tumor markers showed an increased level of carcinoembryonic antigen (CEA). Gastroscopy revealed a neoplasm in the gastric antrum. Specifically, a lump around the annulus of the gastric antrum was observed as well as the formation of a surface ulcer, white coating, invasion of the pylorus, and no abnormalities in the duodenal bulb and descending part. Subsequent pathohistological examination of the biopsy showed adenocarcinoma. However, she had no symptoms such as nausea and abdominal pain. Without distant metastases, the patient (cT3N2M0, III) underwent radical gastrectomy for distal gastric cancer in Shandong Cancer Hospital and Institute in October 2018. Postoperative pathological staging was T3N2M0 IIIA, according to the eighth version of American Joint Committee on Cancer/Union for International Cancer Control (AJCC/UICC) TNM classification of gastric cancer. The pathohistological results of gastric cancer resection showed that the mucinous adenocarcinoma had invaded the muscularis propria and reached the subserosal layer in the distal stomach. No cancer was found in the upper and lower tangents. Regional lymph node status was as follows: lesser curvature side (2/12), greater curvature side (1/7), “1st group” (0/2), “4th group” as adipose tissue, “7th, 8th, 9th group” (2/8), “11th group” (0/3), and “12th group” as adipose tissue. There was no carcinoma in the omentum. Immunohistochemistry showed that human epidermal-growth-factor receptor 2 (HER-2), Epstein–Barr virus (EBV), and tumor suppressor gene P53 were not expressed, whereas MLH1, PMS2, MSH2, and MSH6 were expressed in the tumor. The microsatellite state was stable. However, the patient did not accept postoperative adjuvant therapy because of personal reasons. In June 2019, the patient began to feel dizzy, and multiple brain metastases as well as multiple axillary lymph node metastases were found by computed tomography (CT). Apart from those, CT did not show signs of other metastasis, such as in the lungs, pancreas, spleen, and gallbladder. The patient (rpT3N2cM1, IVb) accepted systemic chemotherapy. As the first-line palliative chemotherapy, she received one course of oxaliplatin 130 mg/m^2^ combined with tomudex 3 mg/m^2^. Afterward, the patient received whole brain radiotherapy (WBRT), which consisted of 2 Gy once daily in 20 fractions adding up to a total dose of 40 Gy. After 20 Gy of radiation, the patient showed patchy ecchymoses over limbs on July 7, 2019, without any traumatic injury, fever, nausea, vomiting, diarrhea, or melena. The level of fibrinogen declined progressively to 0.39 g/L (normal value, 2.00–4.00 g/L; the critical value, <1 g/L), while there was no significant abnormality in her blood counts and other coagulation indicators. Relevant coagulation-related indicators were as follows: platelets 110 × 10^9^/L (reference value: 125–350 × 10^9^/L), thrombin time 20.2 s (reference value: 11–17 s), prothrombin time (PT) 13.4 s (reference value: 8.8–13.8 s), activated partial thromboplastin time (APTT) 32.9 s (reference value: 26–42 s), D-dimer 2.41 mg/L (reference value: 0–1 mg/L), and fibrinogen degradation product 33.05 μg/mL (reference value: 0–5 μg/mL). Infusion of fresh frozen plasma, cryoprecipitate, and fibrinogen concentrate was not effective, and reexaminations of fibrinogen still indicated the critical value, as shown in Figure 1A. Therefore, WBRT was discontinued on July 13, 2019, at the cumulative dose of 32 Gy. Subsequently, the patient was transferred to our hospital for further treatment.

**FIGURE 2:**
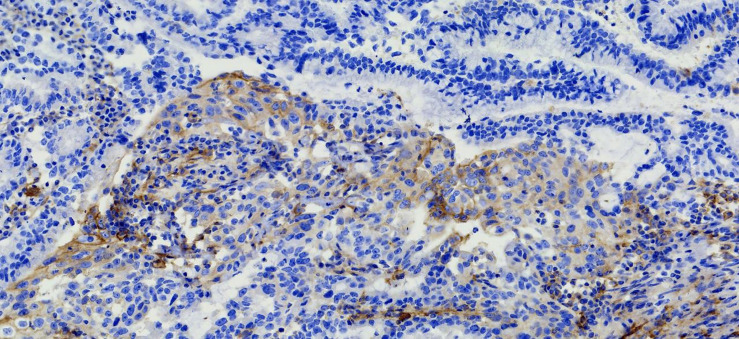
Immunohistochemical PD-L1 staining (Magnification: 10 × 40). The patient’s expression level of PD-LI was detected using the *VENTANA PD-L1 (SP263) Assay*. The brown cells in the immunohistochemical staining for PD-L1 are positive cells, including tumor cells (TCs) with membrane staining at any intensity and immune cells (IC) in the tumor area, such as lymphocytes and macrophages. After counting the positive cells, the corresponding proportion was calculated according to the formula. Tumor Proportion Score (TPS), which is the percentage of viable tumor cells showing partial or complete membrane staining at any intensity. Combined positive score (CPS) = (positive TC + positive IC)/total tumor cells × 100.

**FIGURE 3:**
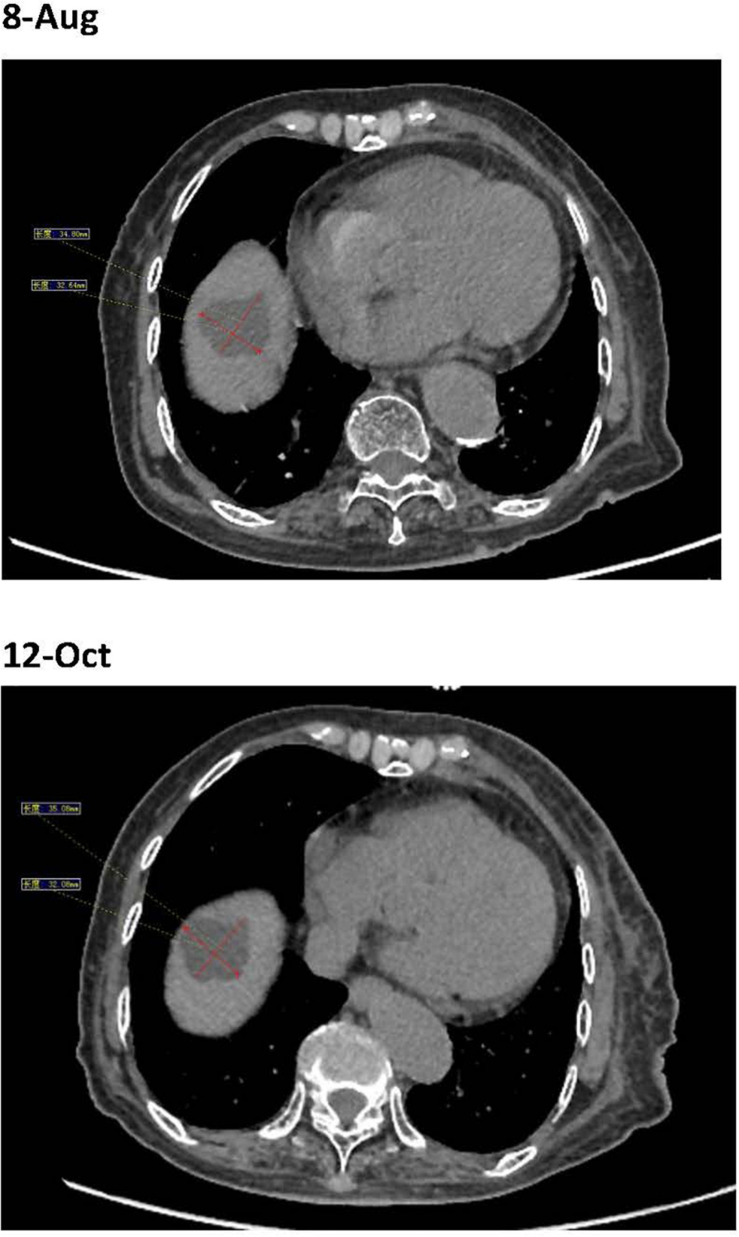
Before the fourth immunotherapy by sintilimab, the liver metastases showed no obviously change.

The patient’s past medical history included hypertension, cerebral infarction, and coronary artery disease. She had no family history of bleeding or clotting disorder. Physical examination was normal apart from the multiple red ecchymoses of irregular shapes, fresh or old on the limbs’ skin. Blood biochemistry and renal and hepatic function tests were within normal limits [albumin 40.6 g/L (reference value: 40–55 g/L), AST 21.1 U/L (reference value: 13–35 U/L), ALT 32.4 U/L (reference value: 7–40 U/L), and total bilirubin 8.2 μmol/L (reference value: 3.4–17.1 μmol/L)]. We continued fibrinogen replacement therapy and administered vitamin K1 to prevent severe spontaneous hemorrhage, such as cerebral and gastrointestinal hemorrhage. Although cryoprecipitate, fibrinogen concentrate, and plasma were given more than a month in total, hypofibrinogenemia persisted. In addition, abnormally high serum concentrations of CEA continued to rise (Figure 1C). Computed tomography indicated multiple metastatic tumors in the S3 and S8 segments of the liver and 3.3-cm-long metastatic lymph nodes. Therefore, we tried to treat her primary disease. She could not tolerate systemic chemotherapy, as she was too weak to take care of herself. Craniocerebral magnetic resonance indicated no intracranial metastases after the radiotherapy. Considering the previously detected indicators of this patient, such as tumor proportion score (TPS) > 15% and combined positive score (CPS) of 20, the expression of programmed cell death 1 ligand 1 (PD-L1) was considerable (TPS > 1%, CPS > 1) on tumor cells (TCs), immune cells, and mesenchymal cells (Tables 1, 2 and Figure 2). Therefore, immunotherapy was deemed a feasible and effective choice for her primary disease. She started receiving sintilimab, 200 mg per time, every three weeks from August 17, 2019. After the first immunotherapy, fibrinogen level increased to 1.24 g/L. Before the fourth immunotherapy, fibrinogen rose to 1.98 g/L, a near-normal value (Figure 1B). Computed tomography of the chest and abdomen suggested that the therapeutic effect was stable disease (SD) on October 12, 2019 (Figure 3), according to Response Evaluation Criteria in Solid Tumors (RECIST Version 1.1). The liver metastases showed no obvious change (Figure 3), and the left axillary lymph node was smaller than before (25 mm vs. 33 mm). CEA decreased from 803.9 to 110 ng/mL (Figure 1C). At the time of the fifth immunotherapy, the patient showed some symptoms, such as increased stool frequency, loose stool, and fatigue, which were considered to be the adverse effects of sintilimab. The treatment of sintilimab continued until May 5, 2020, when the fibrinogen level dropped back to 0.48 g/L. The patient achieved progression-free survival for nearly 8 months.

**TABLE 1:** PD-L1 test results.

Test content	Results	
Total number of gene mutation	8	
Total number of gene mutation related to drugs	0	
TMB	7.01/Mb	
Pd-L1 expression	TPS:20%	CPS:30
MSI	MSS	
Germ line pathogenic variation	0	Suspected variation:0

*The detection was a full-target gene detection of solid tumor, covering the exon regions of 457 genes and detecting four types of mutations including SNV, InDel, Fusion, and CNV. It also includes immunotherapy markers, such as TMB, MSI, PD-L1 expression levels and DDR system-related genes. Pd-L1, programmed cell death 1 ligand 1; TMB, tumor mutation burden; TPS, tumor proportion score; MSI, microsatellite instability; MSS, microsatellite stability; SNV, single nucleotide variation; CNV, copy number variation; DDR, DNA Damage Repair.*

**TABLE 2:** The eight gene mutations of the patient.

Gene	EXON	Mutation type	Mutation abundance (%)
BRCA1	10	SNV	27.71
Fam46C	2	SNV	6.62
FGF14	1	SNV	10.54
FGFR4	18	SNV	26.71
PTPRT	9	SNV	5.29
TP53	4	SNV	29.09
XPO1	25	SNV	4.32
FBXW7	7	Deletion	17.01

## Discussion

In general, tumor patients are prone to hypercoagulability and have a high risk of thrombosis. Our patient was no exception at the beginning. She was first treated for venous thrombosis of the right lower extremity, which led to the screening for gastric cancer. She also had a history of cerebral infarction, another condition possibly associated with hypercoagulability. In regard to gastric cancer and fibrinogen levels, most studies indicated fibrinogen elevation as an indicator of poor prognosis. Previous studies illustrated that fibrinogen might be correlated with the tumor progression of gastric cancer (6), although the mechanism remained unclear.

Gastric cancer concomitant with unprovoked hypofibrino genemia has been rarely reported. After multiple metastases, the patient developed hypocoagulability during antineoplastic therapy, which contradicts common hypercoagulability associated with chemotherapy and supportive care agents. Fibrinogen supplementation alone was not effective in treatment, which suggested that hypofibrinogenemia may be related to the primary disease. This was confirmed by increasing fibrinogen level after immunotherapy. We decided to apply immunotherapy for the primary disease in our case due to several reasons. Namely, prospective studies supported the use of immunological checkpoint inhibitors, and PD-1 monoclonal antibody has already been approved in the United States and Japan as the third-line treatment for advanced gastric cancer (7). Recent studies have suggested that mismatch repair deficiency (dMMR) and EBV positivity are molecular indicators for immunotherapeutic benefits (8). The mismatch repair (MMR) system consists of several proteins including the products of the MLH1, MSH2, MSH6, and PMS2 genes, and it is responsible for the surveillance and correction of errors in DNA replication. The dMMR means a failure to correct errors, which results in a strong “mutator phenotype” with numerous frameshift mutations in coding and non-coding microsatellites (9). Our case seemed to respond well and her blood parameters improved during immunotherapy though she was in microsatellite stability (MSS) and negative for EBV. Immunotherapy is a new approach that is still in the exploratory stage; hence, it is important to further explore the molecular characteristics of patients with advanced gastric cancer who are sensitive to immunotherapy.

Considering the economic situation of the patient, we chose the domestic drug sintilimab. The added effect of improved coagulation was amazing. The mechanism of hypofibrinogenemia caused by gastric cancer is not clear. There may be several reasons for sudden hypofibrinogenemia. Firstly, fibrinogen is an acute-phase protein and its production is regulated by cytokines. For example, interleukin-4 (IL-4) suppresses its production (10). Patients with gastric carcinoma have increased IL-4 level (11), which may interfere with the production of fibrinogen. Further studies are needed to explore whether cytokines modulate fibrinogen level via interaction with each other. We will evaluate IL-4 and other cytokines in the patient’s follow-up visits to verify the relationship between cytokines and hypofibrinogenemia. Secondly, hypofibrinogenemia may stem from impaired liver function, which would be manifested as prolongation of PT or elevation of transaminases (12). However, our patient’s index of liver function was normal, as transaminases and bilirubin were within the normal ranges; therefore, it could not be a reliable explanation for low fibrinogen level in this patient.

There are various factors that may result in hypofibrinogenemia. First of all, congenital hypofibrinogenemia is a type of congenital fibrinogen disorder due to bi-allelic mutations in one of the three fibrinogen genes: fibrinogen alpha chain (FGA), fibrinogen beta chain (FGB), or fibrinogen gamma chain (FGG); these encode α, β, and γ fibrinogen polypeptides, respectively, which are folded together to form the mature fibrinogen (13). Secondly, circulating fibrinogen can be consumed with systemic activation of the coagulation cascade in some diseases. For example, in severe sepsis, antithrombin synthesis is down-regulated and consumption is markedly increased due to ongoing thrombin formation (14). In our case, white blood cell counts and the classification did not show any abnormality, and imaging examinations did not find any signs of infection such as lung infection. Considering that the patient had no symptoms that suggested infection such as fever, we thought it was unlikely that infection was the cause of hypofibrinogenemia. However, specific antigen or antibody tests in the diagnosis and treatment might help exclude the recent infection more accurately. The additional mechanism of hypofibrinogenemia in primary hemophagocytic lymphohistiocytosis (HLH) is uptake of fibrin and/or fibrinogen by activated histiocytes (15). Thirdly, hypofibrinogenemia is an important component of acute traumatic coagulopathy (16). Previous studies have shown that fibrinolytic activity increased in trauma patients after injury, which impaired the ability to control bleeding (17). Another important cause of hypofibrinogenemia may be adverse reactions to drugs, such as reported for tigecycline, hemocoagulase, and sodium valproate (18), which is confirmed by fibrinogen recovery after drug discontinuation. For example, hemocoagulase exerts its hemostatic effect by hydrolyzing the A α chain of fibrinogen; therefore, it can continuously consume fibrinogen and lead to reduced plasma concentration (19). The drugs also affect the level of fibrinogen synthesis-related cytokines, such as IL-4 and interleukin-13 (IL-13), but the specific mechanism needs further study (18). Last but not least, disseminated intravascular coagulation (DIC) is an occasional complication of solid tumors, usually identified by excessive bleeding or abnormal laboratory test results, such as low-PLT, low-fibrinogen, low-clotting factors, and high-D-dimer. As the dominant tumor type associated with DIC is adenocarcinoma (20), our patient clearly had a common type of cancer that is related to DIC. However, platelets in our case did not decrease to 100 × 10^9^/L, and PT and APTT did not shorten initially or elongate subsequently. It has been reported that some cases would only display a moderately decreased platelet count or nearly normal clotting assay results due to adequate compensation for the consumed platelets and coagulation factors (21). Indeed, no laboratory test can strictly rule in or rule out the diagnosis of DIC. In contrast, there is no single laboratory test or combination of tests that can substantiate unequivocal diagnosis of DIC (20). Moreover, DIC in malignant blood disease has unique features characterized by a marked and sometimes excessive activation of endogenous fibrinolysis. This concept has been confirmed by the clinical presentation of major bleeding in combination with laboratory findings of hyperfibrinolysis, that is, very low levels of fibrinogen, sky-high levels of D-dimer or other fibrin degradation products, and a marked deficiency of plasminogen and α2-antiplasmin (22). To sum up, our patient might have had a special type of cancer-associated DIC, or could not be simply diagnosed as DIC by laboratory tests. Nevertheless, in specific cases where hyperfibrinolysis dominates the coagulopathy, such as in adenocarcinoma-induced DIC, antifibrinolytic treatment can be helpful (21), usually by lysine analogs, such as tranexamic acid (23). Disseminated intravascular coagulation typically disappears if the malignancy is effectively treated (24). Our case is consistent with the recovery of clotting problems after effective immunotherapy for the tumor. We also analyzed several possible causes of cancer-causing hypofibrinogenemia in DIC: (a) As many cancers stimulate synthesis and discharge of cytokines or provoke other cells to activate cytokine systems, it may be presumed that inflammatory modulations of coagulation, anticoagulation, and endogenous fibrinolysis are crucial in the development of malignancy-related DIC. Changes in fibrinolysis are usually attributed to tumor necrosis factor-α (TNF-α) (21). (b) Tumor cells can elicit DIC by expressing fibrinolytic proteins. Many tumors can display plasminogen-activating factors, including urokinase-type plasminogen activator (u-PA) and tissue-type plasminogen activator (t-PA), which may contribute to a hyperfibrinolytic condition (25). (c) In addition, radio- and chemotherapy may cause endothelial cell disruption. This may provide a suitable surface for the assembly of a platelet-fibrin clot (21), which causes the consumption of related factors.

However, hitherto there have been no related reports about the effect of PD-1 on blood fibrinogen. While exploring the relationship between immune response and hypofibrinogenemia, we obtained some hints from acquired HLH caused by malignant tumor, a hyperreactive inflammatory disease (26). Similarly, as TCs cannot be effectively eliminated by the body, they persist and affect the balance of immune system. Tumor cells continuously stimulate and activate immune cells, leading to the proliferation of cytotoxic T lymphocytes (CTLs) and macrophages, which fail to play an adequate immune role, further triggering cytokine storm and producing a higher inflammatory response. Among them, activated macrophages secrete more plasminogen activator, which leads to increased level and activity of plasminogen, and consequently to hypofibrinogenemia. PD-1 inhibitor blocks the binding between TCs’ PD-L1 and T lymphocytes’ PD-1, thus weakening the negative regulatory immune response caused by PD-1/PD-L1 binding and strengthening the immune function. The release of a large number of cytokines can be alleviated, and fibrinolytic reaction can be reduced. As a result, the level of fibrinogen improves. If studies can clarify whether there is a more comprehensive and specific cytokine pattern, the profound mechanism will be greatly promoted. Viruses and bacteria can also act as pathogens and cause an immune response. Specifically, in this case, the patient did not have a fever and hepatosplenomegaly, so the possibility of secondary HLH of malignant tumor or infection was considered to be small. However, the way that immune drug improved the level of the coagulation factor through immune response deserves our attention. During the treatment, the patient received radiotherapy and a large number of TCs were killed, which aggravated the local inflammatory response. This may have also been an important factor. The immune network is complex and the radical mechanism needs further experimental research.

Thus, although sintilimab immunotherapy showed a good therapeutic effect on gastric cancer with hypofibrinogenemia in this patient, we were not completely sure of the causal relationship between sintilimab and hypofibrinogenemia recovery. In addition, we did not examine the levels of fibrinogen in biopsy tissue and acute-phase proteins in blood, which might also affect the coagulation. The exact mechanisms of cancer-related hypofibrinogenemia and sintilimab-induced hypofibrinogenemia recovery should be further clarified by future basic and clinical studies.

## Conclusion

This is the first report of sintilimab reversing a rare, refractory hypofibrinogenemia in a gastric cancer patient. Further studies are needed to clarify the mechanisms of hypofibrinogenemia reversion following immunotherapy by sintilimab.

## Data Availability Statement

The raw data supporting the conclusions of this article will be made available by the authors, without undue reservation, to any qualified researcher.

## Ethics Statement

The studies involving human participants were reviewed and approved by Jinan Central Hospital Affiliated to Shandong University. The patients/participants provided their written informed consent to participate in this study. Written informed consent was obtained from the individual(s) for the publication of any potentially identifiable images or data included in this article.

## Author Contributions

SM and MS analyzed the clinical and histological sections and wrote the manuscript. All authors treated the patient and read and approved the final manuscript.

## Conflict of Interest

The authors declare that the research was conducted in the absence of any commercial or financial relationships that could be construed as a potential conflict of interest.

## Abbreviations

WBRT: whole brain radiotherapy
PD-1: programmed death receptor 1
CTLA-4: cytotoxic T-lymphocyte antigen 4
CEA: carcinoembryonic antigen
AJCC/UICC: American Joint Committee on Cancer/Union for International Cancer Control
HER-2: human epidermal-growth-factor receptor 2
EBV: Epstein–Barr virus
CT: computed tomography
TPS: tumor proportion score
CPS: combined positive score
PD-L1: programmed cell death 1 ligand 1
SD: stable disease
RECIST Version 1.1: Response Evaluation Criteria in Solid Tumors
dMMR: mismatch repair deficient
MMR: mismatch repair
MSS: microsatellite stability
IL-4: interleukin-4
FGA: fibrinogen alpha chain
FGB: fibrinogen beta chain
FGG: fibrinogen gamma chain
HLH: hemophagocytic lymphohistiocytosis
IL-13: interleukin-13
DIC: disseminated intravascular coagulation
PT: prothrombin time
APTT: activated partial thromboplastin time
TNF-α: tumor necrosis factor-α
u-PA: urokinase-type plasminogen activator
t-PA: tissue-type plasminogen activator
TCs: tumor cells
CTLs: cytotoxic T lymphocytes
ICs: immune cells
TMB: tumor mutation burden
MSI: microsatellite instability
SNV: single nucleotide variation
CNV: copy number variation
DDR: DNA damage repair.
